# Assessment of an Exhaled Breath Test Using High-Pressure Photon Ionization Time-of-Flight Mass Spectrometry to Detect Lung Cancer

**DOI:** 10.1001/jamanetworkopen.2021.3486

**Published:** 2021-03-30

**Authors:** Shushi Meng, Qingyun Li, Zuli Zhou, Hang Li, Xianping Liu, Shuli Pan, Mingru Li, Lei Wang, Yanqing Guo, Mantang Qiu, Jun Wang

**Affiliations:** 1 Department of Thoracic Surgery, Peking University People’s Hospital, Beijing, China; 2 Shenzhen Breatha Biological Technology Co Ltd, Shenzhen, China; 3 Medical Examination Center, Aerospace 731 Hospital, Beijing, China; 4 Department of Thoracic Surgery, Aerospace 731 Hospital, Beijing, China

## Abstract

**Importance:**

Exhaled breath is an attractive option for cancer detection. A sensitive and reliable breath test has the potential to greatly facilitate diagnoses and therapeutic monitoring of lung cancer.

**Objective:**

To investigate whether the breath test is able to detect lung cancer using the highly sensitive high-pressure photon ionization time-of-flight mass spectrometry (HPPI-TOFMS).

**Design, Setting, and Participants:**

This diagnostic study was conducted with a prospective-specimen collection, retrospective-blinded evaluation design. Exhaled breath samples were collected before surgery and detected by HPPI-TOFMS. The detection model was constructed by support vector machine (SVM) algorithm. Patients with pathologically confirmed lung cancer were recruited from Peking University People’s Hospital, and healthy adults without pulmonary noncalcified nodules were recruited from Aerospace 731 Hospital. Data analysis was performed from August to October 2020.

**Exposures:**

Breath testing and SVM algorithm.

**Main Outcomes and Measures:**

The detection performance of the breath test was measured by sensitivity, specificity, accuracy, and area under the receiver-operating characteristic curve (AUC).

**Results:**

Exhaled breath samples were from 139 patients with lung cancer and 289 healthy adults, and all breath samples were collected and tested. Of all participants, 228 (53.27%) were women and the mean (SD) age was 57.0 (11.4) years. After clinical outcomes were ascertained, all participants were randomly assigned into the discovery data set (381 participants) and the blinded validation data set (47 participants). The discovery data set was further broken into a training set (286 participants) and a test set (95 participants) to construct and test the detection model. The detection model reached a mean (SD) of 92.97% (4.64%) for sensitivity, 96.68% (2.21%) for specificity, and 95.51% (1.93%) for accuracy in the test set after 500 iterations. In the blinded validation data set (47 participants), the model revealed a sensitivity of 100%, a specificity of 92.86%, an accuracy of 95.74%, and an AUC of 0.9586.

**Conclusions and Relevance:**

This diagnostic study’s results suggest that a breath test with HPPI-TOFMS is feasible and accurate for lung cancer detection, which may be useful for future lung cancer screenings.

## Key Points

**Question:**

Is the exhaled breath test feasible and accurate to detect lung cancer using high-pressure photon ionization time-of-flight mass spectrometry (HPPI-TOFMS)?

**Findings:**

In this diagnostic study, alveolar air was collected from 139 patients with lung cancer and 289 healthy participants. The breath test based on HPPI-TOFMS reached a sensitivity of 100%, a specificity of 92.86%, an accuracy of 95.74%, and area under curve of 0.9586 in the validation data set.

**Meaning:**

These findings suggest that an exhaled breath test with HPPI-TOFMS may be a promising approach for lung cancer detection.

## Teaser

This diagnostic study examines whether a breath test that uses high-pressure photon ionization time-of-flight mass spectrometry (HPPI-TOFMS) can accurately distinguish patients with lung cancer from those without.

## Introduction

Lung cancer is the leading cause of cancer-related death worldwide.^1^ Most lung cancer cases are at advanced stages when diagnosed, and only 15% of newly diagnosed lung cancer cases are localized.^2^ Patients with localized lung cancer have a substantially longer life expectancy than those with advanced stage cancer.^3^ Therefore, it is urgent to develop a highly accurate and noninvasive tool for early detection and screening of lung cancer.^4,5^

The National Lung Screening Trial has demonstrated that low-dose computed tomography (LDCT) screening among the high-risk population could reduce lung cancer-related mortality by 20%.^6^ However, LDCT has disadvantages of radiation exposure, high cost, and a high false-positive rate, which may lead to invasive procedures, unnecessary fear of death, and possible adverse events in follow-up.^7,8,9,10^

Exhaled breath may be a better tool for cancer detection because of its noninvasive nature.^11,12,13,14^ Many efforts have been made to develop breath tests that are suitable for lung cancer detection. Gas-chromatography mass spectrometry (GC-MS) is a well-established technique to detect volatile organic compounds (VOCs) in exhaled breath, but the tedious pretreatment steps and time-consuming detection process limit its application.^15^ Electronic noses can be used to perform exhaled breath analysis, but they are not able to quantify the absolute content of VOCs in a mixture.^16^ Direct mass spectrometry, such as secondary electrospray ionization,^17^ selected-ion-flow-tube,^18^ and proton-transfer-reaction,^19^ has been used for rapid detection of exhaled breath; however, the vast amount of water vapor in exhaled breath makes the ionization process more intricate and increases the complexity for data analysis.^20^ High-pressure photon ionization time-of-flight mass spectrometry (HPPI-TOFMS) is a promising tool for breath testing, because it is highly sensitive, does not require pretreatment of exhaled breath, and holds great tolerance for humidity.^21^ HPPI-TOFMS has successfully monitored the concentration of exhaled propofol during surgery and has shown good association with blood propofol concentration and bispectral index.^21,22,23^

As shown by Hanna et al,^14^ previous studies on breath tests have had substantial limitations and potential bias, such as small sample size, poor methodological quality, and lack of validation. In our case-control diagnostic study, we investigated whether a breath test combining HPPI-TOFMS and a support vector machine (SVM) algorithm was able to distinguish patients with lung cancer from healthy individuals.

## Methods

### Participant Recruitment and Study Design

**Figure 1.**
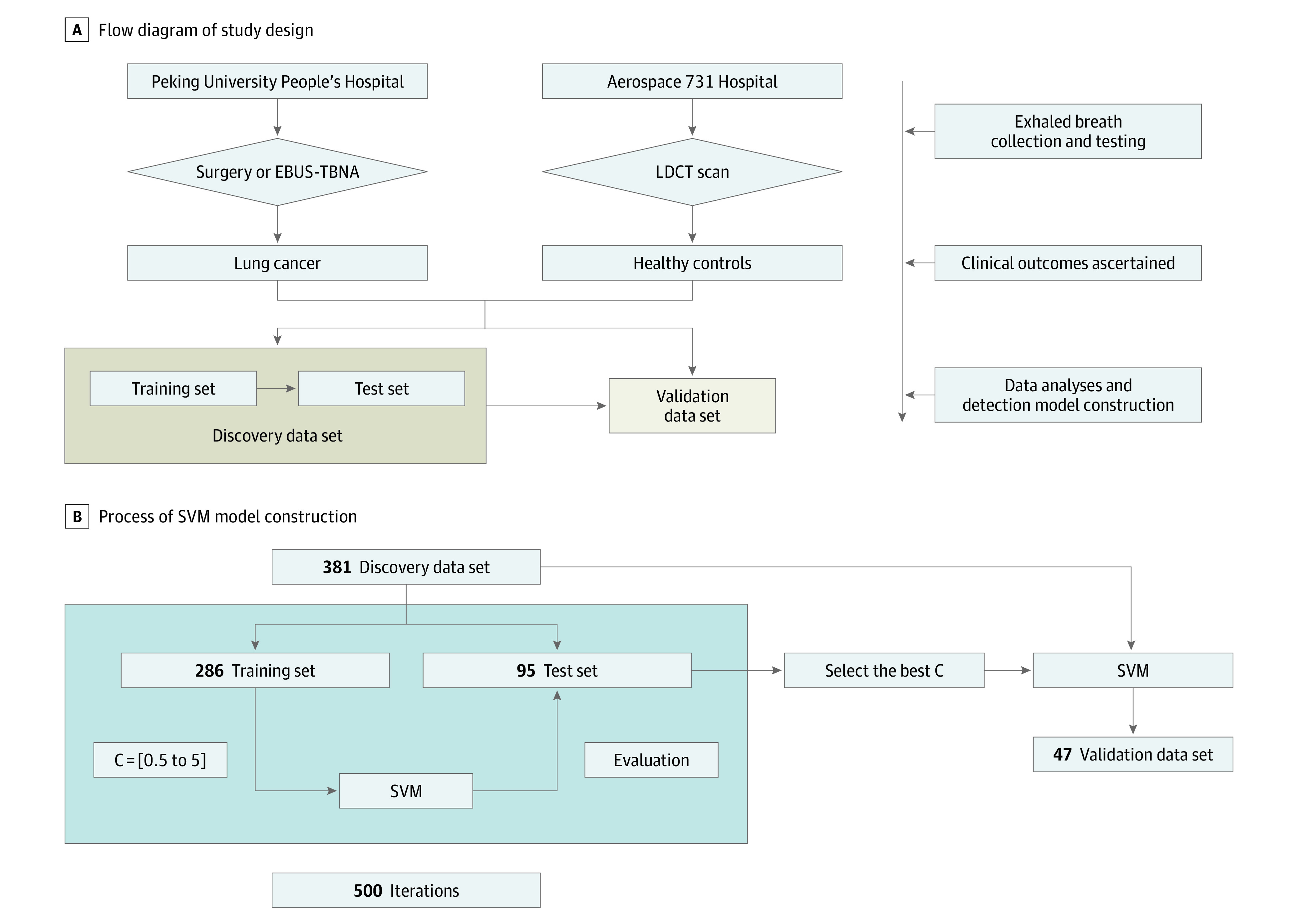
Flow Diagrams of Study Design and the Process of Support Vector Machine (SVM) Model Construction The flow diagrams of study design (A) and the process of SVM model construction (B). C indicates parameter *C*, an important parameter in the SVM algorithm; EBUS-TBNA, endobronchial ultrasonography-guided transbronchial needle aspirate; LDCT, low-dose computed tomography.

This diagnostic study followed the Standards for Reporting of Diagnostic Accuracy (STARD) reporting guideline.^24^ A prospective-specimen collection, retrospective-blinded evaluation (PROBE) design^25^ was used, and the overall study design is shown in Figure 1. This study was approved by the Ethics Committee Board of Peking University People’s Hospital, and written informed consent was obtained from all participants.

Healthy individuals for the control group were recruited from Aerospace 731 Hospital in a population who underwent LDCT for physical examination. Participants were recruited according to the following criteria: (1) aged 18 years or older, (2) no history of cancer within 5 years and no anticancer treatment, (3) no sign of active infections, and (4) no sign of liver or kidney dysfunction. A total of 289 participants without pulmonary noncalcified nodules were selected and served as the control group.

Patients with pulmonary lesions and who had undergone thoracic surgery or endobronchial ultrasonography-guided transbronchial needle aspiration (EBUS-TBNA) were consecutively recruited at the Department of Thoracic Surgery, Peking University People’s Hospital. Patients were recruited with the following criteria: (1) aged 18 years or older, (2) pulmonary lesions seen on computed tomography (CT) images, and (3) plan to undergo thoracic surgery or EBUS-TBNA. Patients who met the following criteria were excluded: (1) history of other types of cancer within 5 years, (2) received anticancer treatment before, (3) pathologically confirmed lung benign diseases, (4) signs of active infections, or (5) liver or kidney dysfunction. A total of 139 patients were identified with pathologically diagnosed lung cancer. For all participants, the clinical data and demographic characteristics were collected from medical records and questionnaires.

### LDCT Examination and Analyses

Spiral CT images were obtained using a 64-detector CT row scanner with a low-dose setting (120 kV [peak], 30 mA) and were reconstructed in overlapping contiguous 5-mm increments at a pitch of 1.25 mm. The LDCT images of all included participants were first analyzed by an artificial intelligence–based program σ-Discover Lung (provided by 12 SIGMA Technology Company, Ltd, Beijing, China), and then confirmed by an experienced radiologist (Y.G.).

### Exhaled Breath Collection

All exhaled breath samples were collected by trained investigators following the same protocol. Exhaled breath samples were collected in the morning for all participants. For patients with lung cancer, it was collected the next morning after admission to hospital. For healthy individuals, exhaled breath was collected at the same day of physical examination and before LDCT examination. All participants fasted for at least 8 hours. To reduce potential confounding factors, all participants were asked not to ingest spicy food, alcohol, or coffee the night before exhaled breath collection.

**Figure 2.**
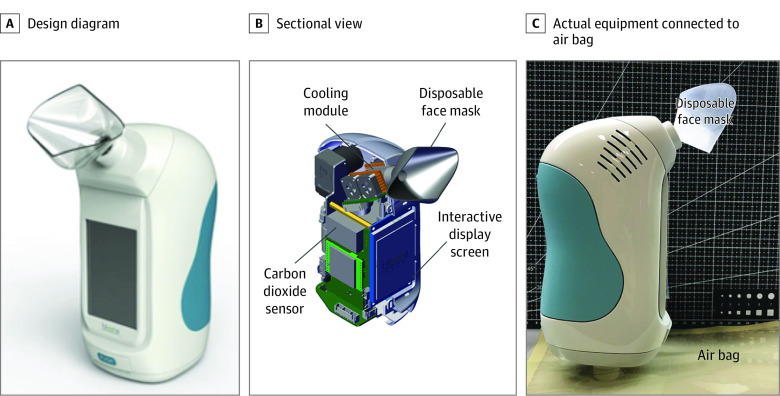
Exhaled Breath Sampling Equipment Images show the design diagram (A) and sectional view (B) of the exhaled breath sampling equipment. The actual breath sampling equipment is connected with an air bag (C).

Home-designed sampling equipment was used for breath sampling, and the breath sample was stored using Tedlar (DuPont de Nemours) air bags. The sampling equipment was composed of a disposable face mask, a cooling module, a CO_2_ sensor, and an interactive display screen, as shown in Figure 2. A disposable face mask was replaced before each collection to avoid cross-contamination. The CO_2_ sensor was used to ensure alveolar air was collected. Briefly, each participant took a deep inhalation and then exhaled slowly through the mask into the apparatus. Exhaled breath collection began once the CO_2_ sensor detected that the CO_2_ concentration exceeded 4%.

Participants first gargled with pure water, then performed a single deep nasal inhalation followed by complete exhalation via their mouth into the air bag. At both clinical centers, breath samples were collected in a fixed room, and the room air was also collected before and after sample collection of participants. All air bags were delivered to the laboratory and tested within 4 hours. Exhaled breath was collected before LDCT scanning for the control group and before surgery or EBUS-TBNA for inpatients. The data analysis team were blind to the clinical diagnosis and clinical team performing surgery were also blind to the breath tests.

### HPPI-TOFMS Detection

The design and structure of HPPI-TOFMS has been reported before.^26^ The HPPI-TOFMS consisted of a vacuum ultraviolet lamp–based HPPI ion source and an orthogonal acceleration time-of-flight (oa-TOF) mass analyzer, and the TOF mass analyzer had a mass resolution of 4000 (full width half maximum) at mass-to-charge ratio (m/z) = 92, which was achieved with a 0.4 m field-free drift tube. The pressure in the HPPI ion source was set at 500 Pa, and 2 capillaries were arranged in the ion source. Gas-phase exhaled breath sample was directly introduced into the ionization region through a 250 μm inner diameter, 0.60 m long stainless-steel capillary from the air bag. To eliminate condensation of exhaled VOCs and minimize possible surface adsorption, the stainless-steel capillary was heated to 100 °C and the HPPI ion source was heated to 60 °C. The TOF signals were recorded by a 400 picoseconds time-to-digital converter rate at 25 kHz, and all the mass spectra were accumulated for 60 seconds. Mass spectrum peaks detected by HPPI-TOFMS with m/z less than 500 were recorded and 32 500 features were extracted from the HPPI-TOFMS data of each exhaled breath sample.

### Detection Model Construction by SVM

The core of this work is to distinguish patients with lung cancer from healthy individuals, which can be treated as a binary classification problem. In machine learning, the binary classification problem can be solved by the regression model or classification model. In this work, we choose the classification model SVM to distinguish between patients with lung cancer and healthy individuals. The core of SVM is to infer the weight *w* and bias *b*, which is treated as the classifier and fixed after training, with the training data set. Given a sample *x*, its prediction score *y* can be obtained by *y* = *w^T^* × *x* + *b*. For the binary classification (ie, patient with lung cancer and healthy individual), the calling threshold is fixed as 0 to classify the positive and negative samples. For example, the sample can be classified as having lung cancer if *y* is less than or equal to 0, or vice versa. For the SVM, the linear kernel is applied to train the model.

For the SVM, the regularization parameter *C* is a critical parameter to constrain a penalty for the misclassified samples. Therefore, we first performed 500 iterations of a 4-fold cross-validation on the whole discovery data set, in which 381 samples were randomly divided into a training set with 286 samples (90 patients with lung cancer and 196 healthy individuals), and a test set with 95 samples (30 patients with lung cancer and 65 healthy individuals). During each training, the sqrt (square root) normalization is applied as the preprocessing strategy to normalize the original data. An SVM model was used to evaluate the mean classification accuracy at 500 iterations for different parameter *C* values. The detection accuracy is unrelated to the parameter *C*. Therefore, we set *C* = 1.0 in this study, which is the default value in the SVM model. By setting *C* = 1.0, the final model was trained and tested on whole discovery data set (381 samples) to construct the detection model and evaluated on the final validation data set (47 samples). The code and supplemental data set have been uploaded to GitHub.

### Statistical Analysis

Sensitivity, specificity, accuracy, positive predictive value, and negative predictive value were calculated to evaluate diagnostic performance of the breath test. Receiver-operating characteristic curve (ROC) and precision recall curve were performed and area under ROC (AUC) was also calculated to evaluate classification performance of the breath test. Baseline characteristics were analyzed with independent *t* test or Fisher exact test. Two-sided *P* < .05 was considered significant. All statistical analyses were performed using SPSS software version 24.0 (IBM) from August to October 2020.

## Results

### Study Overview and Baseline Characteristics

A total of 139 patients with lung cancer and 289 healthy individuals were included in this study. Of all participants, 228 (53.27%) were women, and the mean (SD) age was 57.0 (11.4) years. We collected exhaled breath samples of healthy individuals and patients with lung cancer from March 1 to September 1, 2019. After diagnoses of all participants were ascertained, all samples were randomly assigned to the discovery data set and validation data set. The baseline characteristics of all eligible participants are shown in Table 1. In the lung cancer group, there was a significant increase in mean (SD) patient age (discovery data set: 60.4 [10.5] years for lung cancer group vs 55.7 [12.1] years for healthy control group; *t* = 3.98; *P* < .001). Most patients with lung cancer (126 of 139 patients) were at early stage (TNM stages I and II).

**Table 1.** Baseline Characteristics of Enrolled Participants

Characteristics	Discovery data set			Validation data set		
	Participants, No. (%)		*P* value	Participants, No. (%)		*P* value
	Lung cancer (n = 120)	Healthy control group (n = 261)		Lung cancer (n = 19)	Healthy control group (n = 28)	
Sex						
Male	46 (38.3)	126 (48.3)	.07	9 (47.4)	19 (67.9)	.16
Female	74 (61.7)	135 (51.7)		10 (52.6)	9 (32.1)	
Age, mean (SD)	60.4 (10.5)	55.7 (12.1)	<.001	58.3 (8.5)	53.9 (8.1)	.67
Body mass index, mean (SD)^a^	23.8 (3.5)	24.7 (3.2)	.29	25.0 (2.8)	25.0 (3.3)	.18
Smoking						
Ever	26 (21.7)	56 (21.5)	.96	4 (21.1)	11 (39.3)	.19
Never	94 (78.3)	205 (78.5)		15 (78.9)	17 (60.7)	
Pathology						
Adenocarcinoma	103 (85.8)	NA	NA	19 (100)	0	NA
Squamous cell carcinoma	14 (11.7)	NA	NA	0	NA	NA
Small cell lung cancer	1 (0.8)	NA	NA	0	NA	NA
Others	2 (1.7)	NA	NA	0	NA	NA
TNM stage						
I	97 (80.8)	NA	NA	17 (89.5)	NA	NA
II	12 (10.0)	NA	NA	0	NA	NA
III	9 (7.5)	NA	NA	1 (5.3)	NA	NA
IV	2 (1.7)	NA	NA	1 (5.3)	NA	NA

Abbreviation: NA, not applicable

^a^ Body mass index is calculated by weight in kilograms divided by height in meters squared.

### HPPI-TOFMS Exhaled Breath Tests

All invited participants agreed to participate in the study with a patient acceptability rate of 100%, and we performed successful sample collection for all participants. The exhaled breath collection process took approximately 60 seconds for a participant. No adverse events were observed during breath sample collection.

### Establishment of a Model for Lung Cancer Detection

As shown in Figure 1, 381 participants were randomly assigned to discovery data set, including 120 patients with lung cancer and 261 healthy individuals. The discovery data set was further broken into a training set and a validation set to establish a lung cancer detection model. In the training set of 90 patients with lung cancer and 196 healthy individuals, SVM algorithm successfully established a detection model, and this model could distinguish patients with lung cancer from healthy individuals. Then, this detection model was further examined in the test set consisting of 30 patients with lung cancer and 65 healthy individuals. After 500 iterations, this detection model reached a mean (SD) of 92.97% (4.64%) for sensitivity, 96.68% (2.21%) for specificity, and 95.51% (1.93%) for accuracy in the test set. This detection model was named Breath Detector of Lung Cancer (BreLC) v1.0.

### Evaluation of the Model in the Validation Data Set

**Figure 3.**
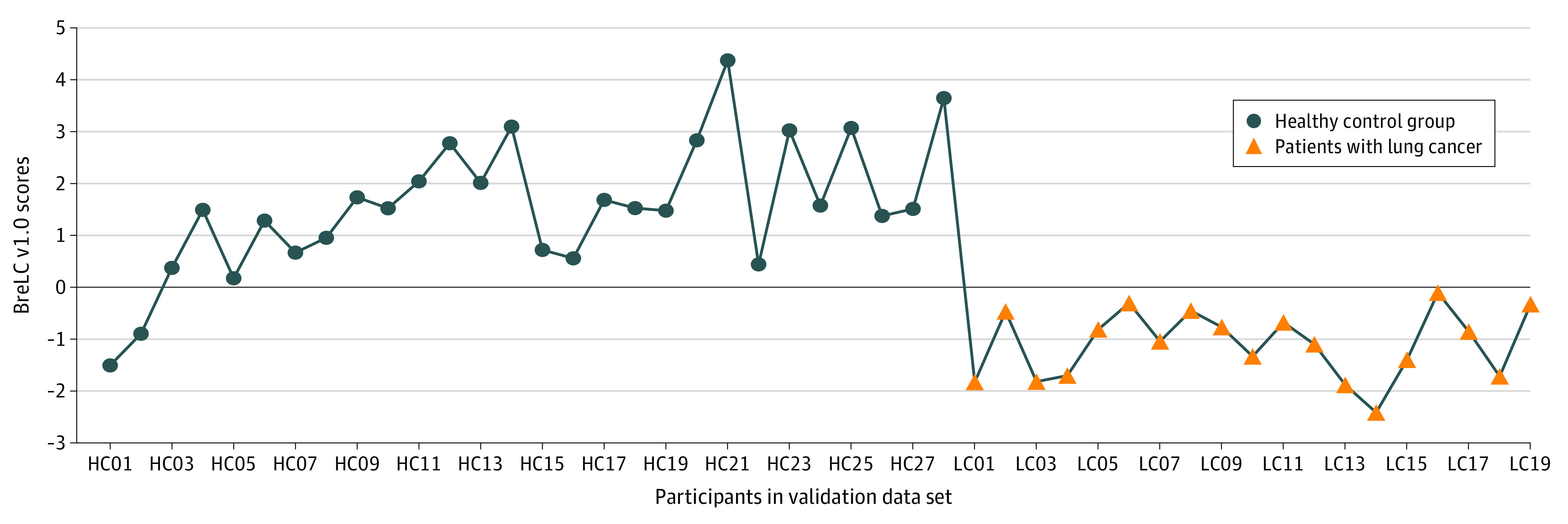
Model Scores of Each Participant in Validation Data Set The validation data set included 47 participants, with 28 individuals in the healthy control (HC) group and 19 patients with lung cancer (LC). Numbers on x-axis refer to participant identification numbers. BreLC indicates Breath Detector of Lung Cancer.

According to PROBE design, 47 participants were assigned to the blinded validation data set. The model revealed a sensitivity of 100%, a specificity of 92.86%, an accuracy of 95.74%, and an AUC of 0.9586 in this blinded validation data set. As shown in Table 2 and Figure 2, all lung cancer cases were correctly detected by the model, yielding a positive predictive value of 90.48% and a negative predictive value of 100%. Scores of all participants are shown in Figure 3. The ROC curve showed that the model reached an AUC of 0.96 in the blinded validation data set, and the precision-recall curve also demonstrated the robustness of the model (AUC of precision-recall curve was 0.94).

**Table 2.** Detection Performance of the Model in Validation Data Set

Model prediction	Clinical outcome, No.	
	Lung cancer	Healthy individuals
Lung cancer	19	2
Healthy controls	0	26

## Discussion

In this study, we demonstrated that the HPPI-TOFMS breath test is feasible in clinical practice. We have also established that a model for lung cancer detection was associated with high sensitivity and specificity.

On the basis of its noninvasive nature and high acceptability, breath testing is considered an improved tool for diagnosis and screening. Many studies have investigated the diagnostic efficacy of exhaled breath for lung cancer.^2,14,27,28^ According to Hanna et al,^14^ these studies had small sample size and low methodological quality. We have made many efforts to overcome these shortages. First, we used a PROBE design in this study. Exhaled breath was collected and tested before LDCT scanning and pathological diagnosis of lung cancer; therefore, the clinical outcome was unknown for the whole research team. After recruitment of all eligible participants, the samples were randomly assigned into the discovery and validation data sets, and the data analysis team was blind to the clinical outcome. By collection and testing breath samples prospectively and analyzing data retrospectively and blinded, we maximally controlled for potential selection bias. Second, the most common bias in biomarker research is the systematic differences between case and control population, and we have improved many methodological details to ensure standardized sample collection and minimize measurement bias. We have (1) set up a sample collection team who were trained and followed the same protocol; (2) designed sampling equipment that included a CO_2_ sensor to ensure alveolar air was collected and minimized individual variances; and (3) exhaled breath was collected in a fixed room in each clinical center and room air was also collected before and after sample collection to reduce environmental factors. Third, a discovery data set and a blinded validation data set for rigorous evaluation of classification accuracy are essential for development of biomarkers,^29,30^ whereas most studies in lung cancer include only 1 stage and they have small sample size. Therefore, the designed validation data set improves the quality and robustness of our study.

Compared with GC-MS, HPPI-TOFMS does not require sample pretreatment or VOC enrichment and it takes only 60 seconds to analyze a sample. HPPI-TOFMS enhances resolution for more precise identification and quantification of VOCs. In addition, HPPI is one of the most powerful and popular soft ionization techniques for online monitoring of trace VOCs, because of its high ionization efficiency, high molecular ion yield, and low degree of fragmentation.^26,31^ These features make HPPI-TOFMS hold potentially great value for clinical application.

The performance of LDCT for lung cancer screening is not satisfactory so far, and the positive predictive value is approximately 8%, according to a systematic review.^32,33^ Although the blinded validation data set was small, the BreLC v1.0 showed a high positive predictive value of 90.48%, which indicates that BreLC v1.0 may be a promising candidate tool for lung cancer screening. Shlomi et al^2^ also reported that volatile organic compounds in exhaled breath could discriminate patients with lung cancer with *EGFR* variant from those harboring wild-type *EGFR* with an accuracy of 83%. This evidence suggests promising clinical application of breath testing. Moreover, most of the characteristic ions of HPPI were molecular ions or quasi-molecular ions with little clusters, which is beneficial for mass spectral interpretation.^34^

### Limitations

Limitations of this study should also be noted. First, different from previous studies with GC-MS or sensors, the electronic nose for example, we developed the BreLC v1.0 model on the basis of features extracted from HPPI-TOFMS data but not VOCs. This disadvantage makes it difficult to replicate the model with other platforms, and we will further refine our mass spectrometry and establish a feasible and VOC-based detection model. Second, the blinded validation data set is small, and the model was not validated with external data from independent clinical centers. Further multicenter clinical studies will be conducted to validate the model.

## Conclusions

This diagnostic study’s results suggest encouraging findings that breath testing may be a reliable approach to lung cancer detection and HPPI-TOFMS may provide fast and precise detection of exhaled breath. Exhaled breath holds promising clinical application in lung cancer screening.
